# Giant neoplastic omental cyst masquerading as ascites: a case report

**DOI:** 10.4076/1757-1626-2-6482

**Published:** 2009-09-16

**Authors:** Ganiyu A Rahman, Adekunle Y Abdulkadir, Samuel A Olatoke, Stanley Uwaezuoke, Ibrahim F Yusuf, Kolawole T Braimoh

**Affiliations:** 1 Department of Surgery, Division of General Surgery, University of Ilorin Teaching HospitalIlorinNigeria; 2 Department of Radiology, University of Ilorin Teaching HospitalIlorinNigeria

## Abstract

**Introduction:**

Cystic lesion of the omentum and mesentery are rare. The incidence of both cyst types has been variously reported to vary from 1/27,000-100,000 hospital admission. Omental cysts occur three to ten times less frequently than mesenteric cyst. Preoperative diagnosis is infrequently made because of lack of characteristic symptoms and signs.

**Case presentation:**

We present our diagnostic and management challenges in a 43-year-old man with an unusually giant omental cyst confirmed as fibrosarcoma at histology. The cyst gave the abdomen an anteroposterior diameter of about 74 cm that could not be penetrated sufficiently by X-ray photons to produce diagnostic image even at maximum attainable output. Patient benefited from surgical excision. The removed cyst contained about 35 litres of fluid.

**Conclusion:**

Neglected omental cysts as in this case may grow to enormous size, undergo malignant transformation and poses serious diagnostic and surgical challenges.

## Introduction

Cystic lesion of the omentum, mesentery and retroperitoneum have been grouped together in the same category by several authors because they are similar embryologically and pathologically [1].

Though the first reported mesenteric cyst was ascribed to Florentine anatomist Benevieni in 1507 [2], it was not until 1852 when Gairdner described the first reported omental cyst [2]. Omental cyst is less common than mesenteric cyst.

The incidence of both cyst types has been variously reported to vary from 1/27,000-100,000 hospital admission [3]. A high index of suspicion is required to make a clinical diagnosis. Barr and Yamashita [4] stated that “preoperative diagnoses are infrequently made because they are not usually considered in the differential.”

We share our experience on the diagnostic and management challenges in an adult with an unusually giant omental cyst, found to be fibrosarcoma at histology in a resource poor environment.

## Case presentation

A 43-year-old man, a Nigerian of Yoruba ethnicity, presented with 6-year history of progressive abdominal swelling associated pain, weight loss and weakness on mild exertion. He had no facial or leg swelling, symptoms suggestive of impaired renal function, jaundice, vomiting and change in bowel habit.

He admitted to a recent history of early satiety. He had attended five different hospitals and had various investigations and treatments at different times for what was thought to be ascites of chronic liver disease before presenting at our centre. The rapid increase in the abdominal swelling and the increasing difficulty/inability to lie or sleeps in the supine position without dyspnoea a month prior to presentation were of great concern to the patient.

On examination, he was wasted, afebrile, anicteric, dyspnoeic and had finger-clubbing grade II but without other stigmata of chronic liver disease. He was well oriented, conscious and alert, and had globally intact cranial and motor functions.

**Figure 1.**
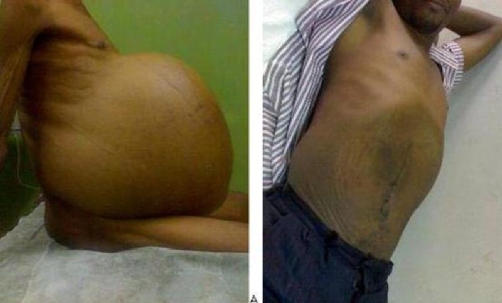
A 43-year-old man with giant neoplastic omental cyst: **(A)** preoperative photograph and **(B)** photograph 6 months after surgery.

Abdomen showed massive non-mobile cystic distension (Figure 1) with positive fluid thrill but not shifting dullness. Liver and spleen were difficult to assess, so was balloting the kidneys. Hernia orifices, external genitalia and digital rectal examination were normal. Clinical differential diagnosis of omental cyst and mesenteric cyst (Probably haemorrhagic cyst) was made.

The patient full blood count, liver function test, fasting blood sugar, Hepatitis B surface antigen (HBs Ag), urinalysis, PT and PTTK were normal. He has had Fine needle aspiration of the liver for cytology in one of his previous hospital visits and it was normal. Abdominal ultrasonography showed intra thoracic displaced normal sized liver with no focal intra-parenchymal lesion, bilateral hydroureteronephrosis worse on the right and a huge multiseptated cystic mass having some scattered mobile bright internal echoes (presumed to be haemorrhages) and irregular solid components. There was neither ascites nor lymphadenopathy. Sonographic impression of mesenteric cyst or omental cyst with suspected malignant transformation was made. Plain abdominal radiograph attempted could not penetrate the mass for diagnostic image even at maximum output attainable due to the size of this mass having an anteroposterior diameter of about 74 cm.

Patient was planned for exploratory laparatomy but he defaulted to represent six months later in respiratory distress, whence he was cachetic, could no longer lay supine for a month, and anaemia had occurred (PCV = 22%.). Ultrasonographic findings this time was still similar to the former except for the increased and aggregating internal echoes presumed to be blood clots. Hence, an impression of haemorrhagic complex intra abdominal cyst (?mesenteric, ?omental) with probable malignant transformation was made.

**Figure 2.**
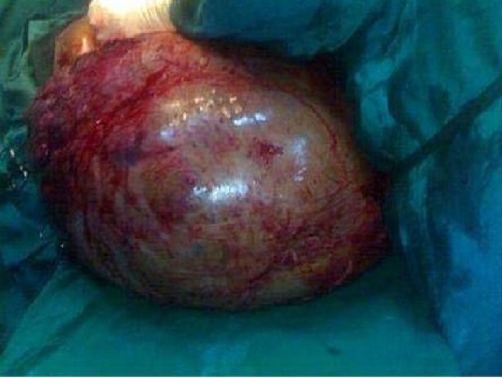
Intra-operative photo showing the giant omental cyst excised. This cyst contains about 35 litres of fluid and weight 37 kg.

He had two pints of blood transfused and emergency exploratory laparatomy under general anaesthesia with endotracheal intubation. Findings at surgery were huge distended abdomen with dilated, tortuous vessels on the anterior abdominal wall and on the parietal peritoneum, huge omental cyst, containing about 35 litres of brownish fluid, and some solid components with areas of necrosis. The mass displaced the stomach and liver upwards, the transverse colon inferiorly, spleen laterally to the left and was adherent to the anterior abdominal wall. There was minimal ascites. He had complete excision of the cyst, which contained 35 litres of brownish fluid and weighed 37 kg (Figure 2).

Anaesthesia was maintained with 100% 0_2_ + Halothane, muscle paralysis with intravenous (IV) 100 mg suxamethonium for intubation then maintained with 6 mg bolus pancuronium followed by intermittent IV 2 mg pancuronium. Halothane was discontinued intraoperatively because of low blood pressure. Anaesthesia was reversed with IV 1.2 mg atrophine and IV 25 mg Neostigmine.

Intraoperatively patient had 10 litres of 0.9% (isotonic) Saline, 4 pints of blood and 10 mls of 10% calcium gluconate. Urine output was 500 mls over the 2 hours that surgery lasted. He was transferred to the intensive care unit (ICU) for monitoring and elective mechanical ventilation. While at the ICU, he had increasing abdominal distension that yielded frank blood on abdominal tap.

He was re-explored and about 1.2 litres of free intra-peritoneal blood oozing from engorged parietal paritoneal vessels was evacuated. There was no area of active visceral bleeding seen. Haemostasis was secured with suture ligation of the oozing parietal peritoneum vessels, through and through apposition of the laxed anterior abdominal wall with sutures and application of abdominal pack and gauze on the anterior abdominal wall held with crepe bandage for tamponade effect. Further 8 litres of isotonic saline and 6 pints of fresh whole blood were given and intravenous calcium gluconate was repeated. Post-operative analgesics and antibiotics were commenced. Vital signs such as respiratory rate, pulse rate and blood pressure improved considerably within 6 hours. He was extubated 16 hours post re-exploratory surgery and continued on intranasal oxygen. By the second post-operative day, he was stable enough and he was commenced on oral intake. He was returned to the surgical ward on the third postoperative day.

Histopathology of the removed specimen showed omental fibrosarcoma. Patient has been follow-up for 8 months and he remains well (see postoperative photograph 6 month later in Figure 1B) and had no evidence of recurrence.

## Discussion

The term mesenteric and omental cysts are merely descriptive topographic site and gross appearance These lesions though uncommon, elicit interest because of their unclear pathogenesis and confusing terminology [5].

Mesenteric cysts have been estimated to be three to ten times more common than omental cysts [6]. However, based on the numbers of reported cases, the ratio may be set at 4.5:1 [6].

According to Conzo *et al* [7], mesenteric and omental cysts are congenital abdominal lesions. However, most reported cases as in this presentation occurred in adult, while only about one third of cases are reported in children younger than 15 years [8]. Probably, the benign nature of these cysts, their generally asymptomatic nature unless when complicated and the non-hindrance on patient day to day activities makes affected patient not to present for medical attention until gross abdominal swelling had set in. Thus, neglected omental cysts as in our case may continue to grow in size and even undergo malignant transformation.

Malignant change has been reported, but is uncommon. Hardin and Hardy [3] reported that malignant transformed omental cysts are usually of low-grade sarcomas and carry a good prognosis if properly excised. Therefore, a complete resection is mandatory because of the high incidence of relapse [7].

Omental masses or cysts lack characteristic specific symptoms and signs. Hence, diagnoses are often not made untill the lesion has attained a size sufficient enough to be palpable or cause compression on organs or neighbouring structures. The diagnosis can therefore be challenging and a high index of suspicion is required [8].

Omental cysts are usually differentiated from ascites by the fact that it is not associated with flanks bulging during recumbency since the cyst will follow as the patient moves. This feature could not be elicited in our patient because of the giant size of the mass at presentation.

Though plain abdominal radiograph and upper gastrointestinal series could be helpful in making diagnosis, the imaging modality of choice is ultrasound [8]. Plain abdominal radiograph was not helpful in our patient due to the extreme size of the cyst that resulted in complete attenuation of the X-ray photons even at maximum kilo voltage attainable by our machine.

Ultrasonography in this patient revealed multiseptated cyst having some solid components and features suggestive of internal haemorrhage. The extensive nature of this cyst hinders visualization of the pancreas. Thus, aside operator dependency, giant omental cyst may impose some ultrasonographic drawback. There were peculiar limitations to CT Scan in this patient ranging from non- ready availability, cost where available, the inability of this patient to lie in supine position without respiratory embarrassment, and above all the CT gantry will not admit the patient based on our previous experience with an abdominal mass that was not as giant as this case. We are aware that CT would have efficiently delineated this mass and its relationship to other intra abdominal structures [5].

The goal of surgical therapy is complete excision of the mass, which sometimes may require inclusion of the adjacent structures [9]. However, in this patient complete excision was achieved without resecting the adjacent stomach or transverse colon.

The histological diagnosis showed omental fibrosarcoma. Primary malignancies of the omentum are rare events but they do occur [10]. In our patient, it is uncertain whether the cyst was malignant ab initio or is a malignant transformation in an omental cyst. The later is known to occur in adult and it appears more probable in this case in view of the prolong history of swelling and the sudden but rapid increasing size of the swelling at later time.

The diagnosis in this patient was very challenging in view of the limited resources. The patient responded well to surgical intervention though he had a turbulent immediate postoperative period.

## Conclusion

The management of this patient with giant omental cyst was challenging but the patient benefited from surgical excision though the immediate postoperative condition was turbulent. Histology of the removed specimen showed fibrosarcoma.

## References

[bib-001] Berger L, Rothenberg RE (1939). Cvsts of the Omentum, Mesentery, and Retroperitoneum. Surgery.

[bib-002] Gairdner WT (1851). A remarkable cyst in the omentum. Transactions of the Pathological Society of London.

[bib-003] Hardin WJ, Hardy JD (1970). Mesenteric Cysts. Am J Surg.

[bib-004] Barr WB, Yamashita T (1964). Mesenteric Cysts. Am J Gastroenterol.

[bib-005] Ross PR, Olmsted WW, Moser RP, Dachman AH, Hjermstad BH, Sobin LH (1987). Mesenteric and Omental cyst: Histologic classification with imaging correlation. Radiology.

[bib-006] Saxena AK Mesenteric and Omental cyst. http://emedicine.medscape.com/article/938463-overview.

[bib-007] Conzo G, Vacca R, Grazia Esposito M, Brancaccio U, Celsi S, Livrea A (2005). Laparoscopic treatment of an omental cyst: a case report and review of the literature. Surg Laparosc Endosc Percutan Tech.

[bib-008] Rahman GA, Johnson A-WBR (2001). Giant Omental Cyst simulating ascites in a Nigerian Child: case report and critique of clinical parameters and investigative modalities. Ann Trop Paediatr.

[bib-009] Vanek VW, Phillips AK (1984). Retrosternal mesenteric and Omental cyst. Arch Surg.

[bib-010] Pfitzmann R, Klupp J, Krenn V, Neuhass P (2004). A dermoid cyst in the greater omentum as a rare epigastric tumor. Z Gastroenterol.

